# Noise and accustomation: A pilot study of trained assessors’ olfactory performance

**DOI:** 10.1371/journal.pone.0174697

**Published:** 2017-04-05

**Authors:** Johanna Trautmann, Lisa Meier-Dinkel, Jan Gertheiss, Daniel Mörlein

**Affiliations:** 1 Department of Animal Sciences, Meat Science Group, University of Göttingen, Göttingen, Germany; 2 Institute of Applied Stochastics and Operations Research, Clausthal, University of Technology, Clausthal-Zellerfeld, Germany; Tokai University, JAPAN

## Abstract

While recent studies suggest an influence of noise on olfactory performance, it is unclear as to what extent the influence varies between subjects who are accustomed to noise and those who are not. Two groups of panelists were selected: a University panel usually working under silent conditions and an abattoir panel usually working on the slaughter line with abattoir noise. Odor discrimination, odor identification, and odor detection thresholds were studied. Furthermore, a sensory quality control task using 40 boar samples was performed. All tests were accomplished both with and without extraneous noise recorded at an abattoir (70 dB) using headphones. Contrary to the researchers’ expectations, abattoir noise hardly affected the olfactory tests nor was the quality control task impaired. Abattoir noise did not influence the perceived intensity of boar taint and the classification results of the testers, regardless of whether they were accustomed to such noise or not. The results indicate that sensory quality control can be conducted in a manufacturing environment with constant noise without diminishing the assessors’ performance.

## Introduction

The main goal of this paper is to evaluate the effect of noise on the olfactory performance of human subjects. We applied standardized odor detection threshold and odor identification tests as well as a more complex odor evaluation task—the latter of which is routinely applied in quality control of foods using the human sense of smell. We decided to involve two groups of trained assessors because these are commonly used for such sensory quality control tasks. The two groups of assessors differed in their previous experience with the tests as well as with their previous exposure to the technical noise applied in our study that seeks to find an effect of accustomation. The results are especially relevant for those involved in sensory science but also for those dealing rather fundamentally researching the sense of smell.

Our decision to focus on olfactory performance as affected by noise stems from the recommendations for good sensory practices stating that a sensory facility should be located away from areas with extraneous noise [1]. In several routine sensory quality control situations assessors evaluate odors/flavors in the presence of various process noises. These situations can occur in the manufacturing environment where sensory assessment is needed during the processing itself. According to European *Directive 2003/10/EC—Noise*, there is an exposure limit value of 87 dB for noise in manufacturing environments. Regardless of legal regulations, noise can occur and is widely defined as unpleasant/unwanted noise. Already in 1968, Holt-Hansen assumed the existence of cross-modal correspondence between taste/flavor and the pitch of a sound according to his results with two kinds of Carlsberg beers and a signal generator [2]. A number of studies have, indeed, investigated the effect of noise on various aspects of human health (e.g.,: [3]–[5], and documented the existence of cross-modal correspondence between sound and taste or odor [6]–[8]. In 1993, the so called Mozart effect has been demonstrated by Rauscher et al.: subjects showed improved performance on an IQ test while listening to music by Mozart [9]. In 2011, Seo and colleagues studied whether the Mozart effect also occurs during an olfactory discrimination task that also highly demands cognitive function [10]. In contrast to Rauscher et al., there was no significant effect of Mozart music on the odor discrimination task. The same study also analyzed whether two different types of background noises exert an influence on odor discrimination tasks: verbal (comedian speaking) and nonverbal noise (din from people at a crowded party) were compared to a silent condition [11]. Subjects performed best in the discrimination task under the silent condition. A comparison of the two noisy conditions revealed that subjects performed worse while listening to a comedian talk than while listening to party noise. These results indicate that the kind of distracting sound is decisive for a potential effect on olfactory performance: constant noise appears less disturbing as people can adapt to it compared to verbal noise which demands more attention. Additionally, a recent study by Walliczek-Dworschak et al. revealed that nonverbal background noise lowered overall olfactory performance while concurrent feedback modulated threshold performance [12].

The question persists whether a noisy environment truly impairs a sensory quality control task.

Such testing situation is that of boar taint evaluation where assessors’ heat and sniff pig carcasses within a noisy slaughter line. In order to perform such tasks, human subjects are carefully selected and then trained over a longer period of time. This olfactory evaluation of boar carcasses is deemed necessary to prevent consumer complaints that else might occur due to tainted pork. Increasing public demands for improved animal welfare forced the European pork production chain stakeholders to declare a ban on surgical castration by 2018 (Declaration of Brussels, 2008). When they reach puberty, the metabolism of intact male pigs leads, especially in fat tissues, to the pronounced accumulation of androstenone and skatole, which may impair consumer acceptance (e.g.,: [2]–[4]). This is the rationale behind our approach to use these two substances in our standardized olfactory tests. In addition, biological (boar fat) samples containing various amounts of these substances were used for a more complex quality control task.

Our decision to involve both a group usually working in a silent environment (University sensory laboratory) and a group usually working in a noisy slaughter plant arose from the question whether the detrimental effect, if any, of noise on olfactory performance was lower when assessors are accustomed to it. To the best of our knowledge, this question has yet not been studied.

## Materials and methods

Informed consent was obtained from all participants and the procedures were approved by the Ethical Committee of the Georg-August-University Göttingen, Germany.

### 2.1 Participants

Twenty trained sensory assessors between 26 to 55 years participated in this survey. They formed two panels consisting of 10 assessors each in accordance with the ISO 8586 sensory analysis guideline, which recommends that a sensory panel should be comprised of at least 10 selected assessors. The university Panel was from the Laboratory for Sensory Analysis and Consumer Research at the University of Göttingen. Its members were carefully selected on the basis of their individual olfactory towards androstenone and skatole [13],[14] and then trained for sensory quality control of boar fat for a period of 8 months up to 3.5. The abattoir panel consisted of staff members from a slaughterhouse, who work in the daily production process controlling the quality of boar carcasses on-line. The university panel is accustomed to a silent working environment, whereas the abattoir panel is accustomed to a noisy environment for sensory evaluations. An audiogram was recorded for the members of the University panel and no auditory disorder was detected thereby. Such tests were, however, not possible for the abattoir panel. Yet, from conversations with these panelists, normal hearing was concluded.

### 2.2 Auditory and olfactory stimuli

For auditory stimulation, tests were accomplished with and without extraneous noise recorded at an abattoir (70 dB); noise was provided using a laptop computer and headphones (Logitech h390). The auditory stimulus was recorded (Audiorecorder ZOOM H2n) for 35 minutes at an industrial slaughter line. It consists of a rather continuous noise schedule caused by the moving slaughter chain with few intermittent yells (staff) or bumps from steel carcass hooks. For each test the assessors were instructed to wear headphones through which, depending on the study design, abattoir noise was or was not played.

Olfactory stimuli consisted of two odor substances related to so-called “boar taint”, i.e., androstenone (5α-Androst-16-ene-3-one) and skatole (3-Methylindol) in various concentrations. They were presented on filter paper strips stored in plastic test tubes and were identified with three digit codes. For both substances a series of 23 binary dilutions (1:1) in propylene glycol (PG) was prepared; starting from level -3 containing 2179.2 μg/g androstenone or 1049.36μg/g skatole (= 8mmol). Level -3 represents the highest odorant concentration and level 20 the lowest odorant concentration; for more detailed information see [9]. Assessors were instructed to briefly sniff each strip three times (approx. 3 seconds). Both substances were used for odor discrimination and odor identification tests as well as for odor detection threshold assessment [9]. Each test was conducted both with and without abattoir noise. Briefly, in the odor discrimination task (3-AFC) triplets of smell strips were presented in a randomized order. In total four 3-AFC tests were applied resulting in 12 smell strips in total for the discrimination task. To prevent olfactory adaptation and sensory fatigue, androstenone and skatole strips were always presented as the odd sample whereas equal samples contained PG only. The task was to discriminate the odd sample from the two identical samples. The interval between the presentations of consecutive triplets was about 20 seconds. Odor identification was assessed for four different concentrations of both androstenone and skatole, for 1.5% butyric acid and for blanks containing PG; each concentration was presented in threefold repetition resulting in 30 smell strips in total for the identification task. Using a multiple choice task the identification was performed from lists of four descriptors: androstenone, skatole, blank and butyric acid. The interval between two odor presentations was approx. 20 seconds.

The University panel, which had extensively been trained in the standardized application of discrimination and identification tests, conducted these olfactory tests on their own while being supervised by the experimenter to make sure that no data manipulation occurs. For the abattoir panel, which in comparison had not been extensively familiarized with these tests, the odor discrimination and identification tasks were under the guidance of a researcher. Thus it was made sure that test procedures and inter-stimulus intervals were comparable for both groups of assessors.

Odor detection thresholds were assessed using a forced-choice stepwise staircase threshold test starting with the weakest concentration. Triplets were presented in a randomized order, with two containing the solvent (PG) and the third containing the odorant. The blind-folded subjects had to discriminate the odor-containing strip. These olfactory tests differ in their demand for cognitive abilities: odor detection thresholds demand less cognitive function than the odor discrimination or identification task [10].

**Fig 1:**
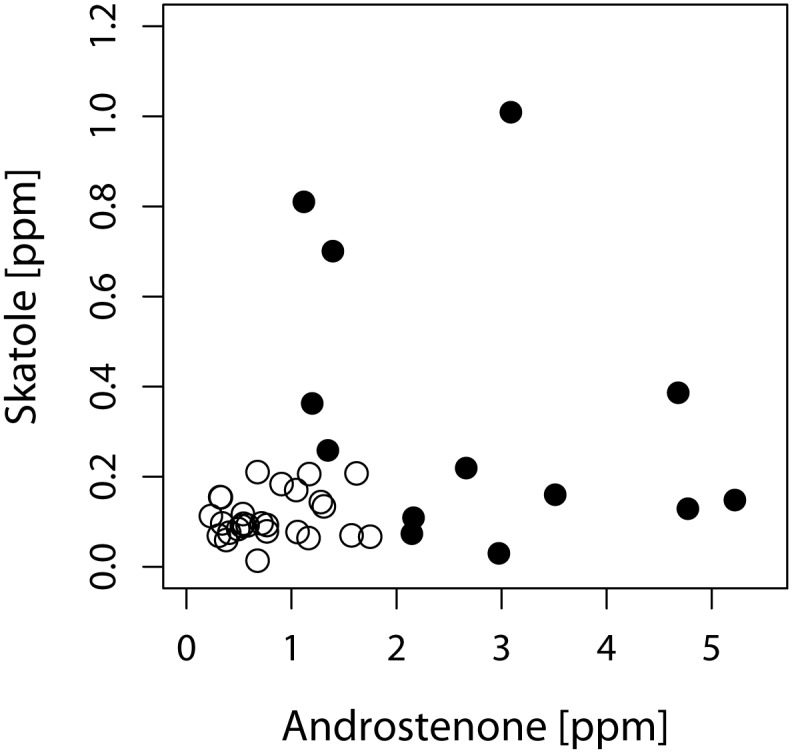
Contents of androstenone and skatole in biological samples of fat tissue of boars used for the quality control task. Boar taint compounds were determined in melted back fat using gas chromatography mass spectrometry. Different symbols indicate whether or not samples are considered to be truly boar tainted (●) or untainted (○) based on chemical analysis as per the definition of CHEMHIGH [21].

Furthermore, 40 boar fat samples with known levels of androstenone (*M* = 1.44 μg/g, range 0.23 to 5.22 μg/g) and skatole (*M* = 0.19 μg/g, range 0.01 to 1.01 μg/g) were evaluated both with and without abattoir noise (Fig 1).

The sample material was derived from a larger study aiming to validate genomic selection for reduced boar taint (STRAT-E-GER Project); for more detailed information see [15]. The samples were taken at a commercial abattoir (Tönnies Lebensmittel GmbH & Co. KG–Home; Long: 8.32240°E; Lat: 51.86262°N). Chemical analysis of boar taint compounds was done in duplicate using gas chromatography mass spectrometry (GC-MS) and deuterated standards [14]; results are given as μg/g (ppm) melted back fat. Samples were presented in a randomized order for each assessor/each condition and identified with a three digit code. A microwave method was used to heat the samples facilitating odorant release, and each panel followed its established sensory quality control protocol. Each panel already had implemented their evaluation system for boar taint in order to cope with their daily evaluation routine. Therefore, each panel applied its individual protocol: the university panel evaluated the samples on a 6-point scale from 0 (=“no deviation from standard fat”) to 5 (=“very strong deviation from standard fat”) [14]. The abattoir panel evaluated the samples on a binary scale (0 = “standard”/no boar taint; 1 = “deviant”/boar tainted). To compensate for the inevitable fact that the two panels used varying protocols/scales, the ratings of the University panel were later dichotomized (see Statistical analysis). The assessors were instructed to sniff three times for approximately 3 seconds; to neutralize their sense of smell they had to sniff their elbows between consecutive samples.

### 2.3 Procedure and test surroundings

The university panel conducted the tests in the Laboratory for Sensory Analysis and Consumer Research at the University of Göttingen. The laboratory was built according to the ISO 8589:2010 norm and has 10 individual booths. The abattoir panel performed the tests in a ventilated room in an administration office at the abattoir. For both panels, the testing environment was nearly silent as would be the case for ordinary sensory lab testing. Discrimination and identifications tests were done on one day with 15 minute breaks; the odor detection threshold tasks were conducted individually for each assessor during four appointments. The fat evaluation was conducted on two consecutive days. For the olfactory and the sensory tests, the subjects were allocated to either the noise or the silent condition according to the randomized experimental design.

## Statistical analysis

All computations were done using the statistical program R [16]. For each panel dependent pairwise t-tests (one-sided) were used to investigate the hypothesis that olfactory performance is impaired under noisy conditions in comparison to silent conditions. In other words, if the odor identification scores are lower, then actual identification was less correct under the noisy condition. Accordingly, if the odor detection thresholds are lower, then the sensitivity level is lower, under the noisy condition compared to the silent condition. Also for the discrimination task one-sided paired t-tests were computed to study whether the performance was significantly impaired under noisy compared to the silent condition. Effect sizes (Cohen’s d) were computed using the *effsize* package in R [17]. To document the reliability of the sensory ratings, intra-class correlation coefficients (ICC; [18]) based on the original ratings of panel U (scores 0 to 5) were computed using the *psych* package [19]. Specifically we were interested in the repeatability of the average rating (consisting of k = 10 raters for each panel) on the same target which is given by the parameter ICC3k. For comparison, we also give the results of the abattoir panel. But those results, in particular confidence intervals, have to be viewed with caution because ICC3k is actually defined for quantitative data, not binary. Furthermore, olfactory test results were analyzed using linear mixed models using the *lmerTest* package [20] applying random subject-specific intercepts and fixed effects for abattoir noise, accustomation, and the interaction thereof. The dependent variables were the numbers of correct identifications (maximum score = 30) or correct discriminations (maximum score = 12), or odor detection thresholds (23 dilution steps).

Also the performance in the sensory quality control task was studied on an individual subject level, based on the agreement between the sensory score and the classification of a fat sample derived from chemically measured key compounds [21]. An agreement/a good performance (coded as 1) means that the sensory rating is consistent with the chemical classification which results in true positive or true negative ratings. No agreement/a bad performance (coded as 0) means that there is no agreement which results in false-positive or false-negative ratings. The sensory ratings of the university panel were converted into a binary variable (original ratings ≥ 2 were recoded into 1 = “boar tainted”; original ratings < 2 were recoded into 0 = “no boar taint”). This was done to compensate for the inevitable fact that the two panels used varying protocols/scales. For chemical boar taint classification the CHEMHIGH thresholds of [21] were used (≥ 2 μg/g androstenone or ≥ 0.25 μg/g skatole). A logistic mixed model (R package *lme4* [22] was used to analyze the factors that may influence the probability of the agreement between sensory evaluation (SENS) and chemical analysis (CHEM), in particular the abattoir noise effect, on an individual level (Eq 1).

$$\text{log }(\frac{\pi_{jkm}}{1-\pi_{jkm}})=\eta_{jkm}=\varphi +u_{j}+v_{k}+n_{m}+a_{m}+(a_{m}*n_{m})+\beta_{1}*AN_{k}+\gamma_{1}*SK_{k}+\;\beta_{2}*AN_{k}^{2}+\;\gamma_{2}*\;SK_{k}^{2}$$ (1)

Here π_jkm_ is the probability of agreement (TN or TP) of a boar fat sample k by assessor j under condition m; φ is the overall mean effect; u_j_ is the random assessor effect; v_k_ is the random effect of the boar fat sample; n_m_ is the fixed effect of abattoir noise; a_m_ is the fixed effect of the accustomation to noise; (*a*_*m*_ * *n*_*m*_) is the interaction effect between abattoir noise and accustomation; *β*_1_ * *AN*_*k*_, $\;\beta_{2}*AN_{k}^{2}$, *γ*_1_ * *SK*_*k*_, and $\;\gamma_{2}*SK_{k}^{2}$ are the fixed effects of the androstenone and skatole concentration of the fat samples. The quadratic terms were included because it can be assumed that the probability of agreement will be higher for both low and high values of androstenone and skatole, but lower for intermediate levels. Such a non-monotone relationship, however, cannot be modeled by a linear effect.

## Results

### 4.1 Effect of non-verbal noise on psychophysical tests

**Fig 2:**
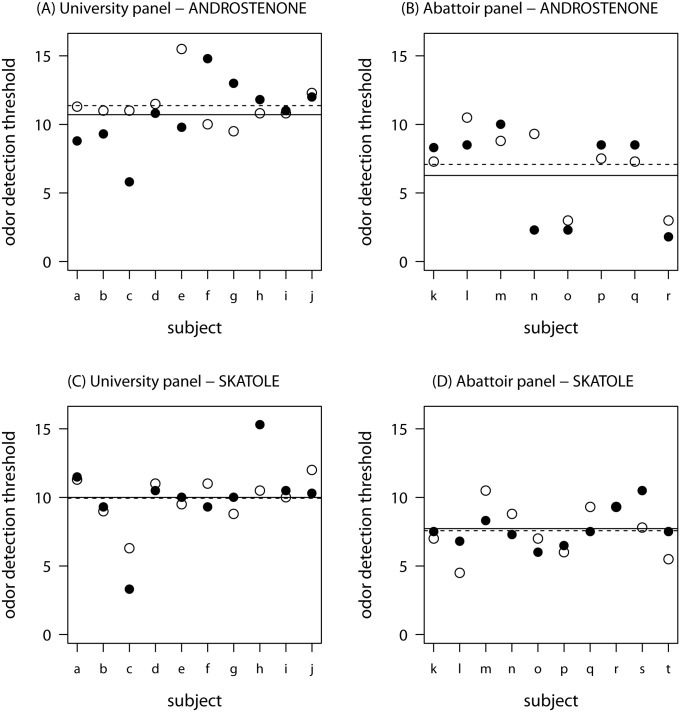
Odor detection thresholds for androstenone and skatole of the university panel (left) and the abattoir panel (right) under noise (●) and silent conditions (○) including the panel average for the silent (- - -) and noisy condition (──). The scores in the threshold test can range between -3 and 20 and they represent a series of binary dilutions of the respective odorant; dilution step -3 corresponds to 8 mMol. That is, a higher dilution step indicates a lower detection threshold, i.e. higher sensitivity. Two assessors of the abattoir panel were obviously insensitive towards androstenone and thus excluded from this analysis. Abattoir noise (70 dB) was applied using headphones.

**Fig 3:**
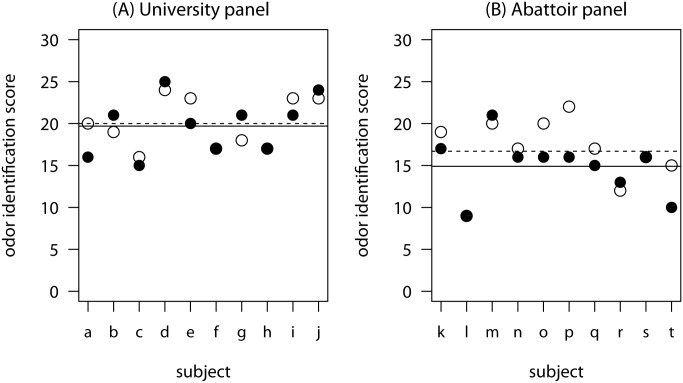
Odor identification task performance of the university panel (left) and the abattoir panel (right) under noise (●) and silent conditions (○) including the panel average for the silent (- - -) and noisy condition (──). The odor identification score ranged between 0 and 30 with the latter score representing a superior performance in identification of various concentrations of androstenone and skatole. Abattoir noise (70 dB) was applied using headphones.

Contrary to the researchers’ expectations, detection thresholds for androstenone were not significantly lower (i.e. assessors less sensitive) under noise compared to silent conditions: for the university panel [*t*(9) = -0.62, *p* = 0.27; *d* = -0.20 (95% CI -1.19 to 0.80)] and for the abattoir panel [*t*(7) = -0.82, *p* = 0.22; *d* = -0.29 (95% CI -1.44 to 0.86)], respectively. Nor was the skatole detection threshold impaired for either the university panel [*t*(9) t = 0.09, *p* = 0.53; *d* = 0.03 (95% CI -0.96 to 1.02)] and for the abattoir panel [*t*(9) = 0.27, *p* = 0.60; *d* = 0.08 (95% CI -0.91 to 1.08)]. However, as the number of assessors and thus the power of the test was comparably low (rarely, trained panels with substantially more than ten assessors are found in reality), also the effect sizes need to be considered. For androstenone odor detection threshold under noisy vs. silent condition, *d* ranged from 0.20 to 0.29 which is deemed a small effect whereas d for skatole detection thresholds (d = 0.03 to 0.08) is deemed not to be relevant. For the identification task, abattoir noise significantly impaired the performance of the abattoir panel [*t*(9) t = -2.29, *p* = 0.02; *d* = -0.72 (95% CI -1.75 to 0.30)] compared to the silent condition. Contrastingly, no significant difference was found for the university panel [*t*(9) t = -0.43, *p* = 0.34; *d* = -0.14 (95% CI -1.13 to 0.86)] between noisy and silent conditions for odor identification. Nor was the discrimination task performance impaired under noise for the abattoir panel [*t*(9) t = -0.13, *p* = 0.45; *d* = -0.04 (95% CI -1.03 to 0.95)] and the university panel [*t*(9) t = -1.41, *p* = 0.10; *d* = -0.14 (95% CI -1.13 to 0.86)], respectively. Individual detection thresholds and odor identification scores as well as the panels’ average performance under noisy and silent condition, are presented in Fig 2 (threshold) and Fig 3 (identification).

### 4.2 Effect of non-verbal noise on sensory quality control of boar fat samples

Neither abattoir noise nor whether assessors were accustomed to that noise (p = 0.48) significantly influenced the agreement between sensory and chemical analyses (see Table 1).

**Table 1:** Parameter estimates of the logistic mixed model using the agreement* between sensory and chemical analysis as response variable for the abattoir and the university panel.

Fixed effects	Estimate (B)	Odds^#^	SE	z value	P[>|z|]
Intercept	0.45	1.57	0.32	1.42	0.16
Condition (0 = silence, 1 = noise)	-0.06	0.94	0.16	-0.39	0.70
Panel (0 = university, 1 = abattoir)	0.17	1.18	0.24	0.70	0.48
Androstenone concentration	-0.25	0.78	0.27	-0.94	0.35
Skatole concentration	-0.57	0.57	1.94	-0.30	0.77
(Androstenone concentration)^2^	0.09	1.10	0.05	1.80	0.07
(Skatole concentration)^2^	4.37	79.42	2.30	1.91	0.06
Condition:Panel (interaction)	-0.13	0.88	0.22	-0.57	0.57

^#^ exp-transformed estimate: = e^[estimate]^

* Agreement is defined as true negative or true positive sensory ratings as compared to the chemical analysis using thresholds for androstenone (≥ 2.0 μg/g) and skatole (≥ 0.25 μg/g melted neck fat)^2^ squared term of concentration

More importantly, no significant interaction between noise and customization to noise was observed with regard to assessor performance based on the logistic mixed model analysis.

**Fig 4:**
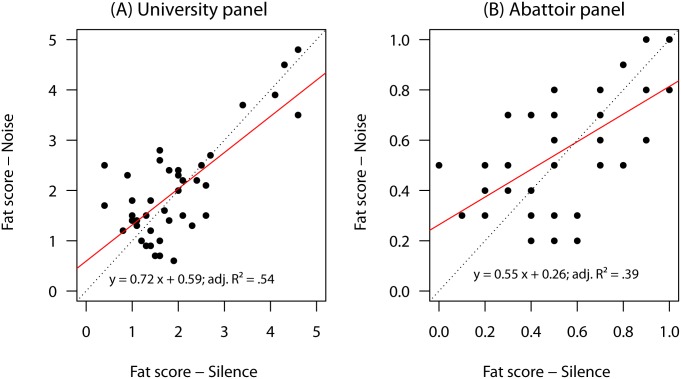
Comparison of averaged fat scores for each sample under noisy and silent condition. For the abattoir panel A the individual fat scores were either 0 (not tainted) or 1 (tainted). That is, the average score over assessors represents a probability that a given sample is tainted. For the University panel U the original scores ranged from 0 (standard pork fat odor) to 5 (strongly deviation from standard pork fat odor). Abattoir noise (70 dB) was applied using headphones.

Also a two-sided paired t-test revealed no significant difference in perceived deviation from standard fat odor (ordinal scores: 0 to 5) in noisy vs. silent conditions for the university panel [*t*(39) = 0.47, *p* = 0.64; *d* = 0.07]. For the abattoir panel, the fat scores per sample (binary scores: 0, 1) were averaged and also subjected to a paired t-test which yielded no significant difference between noisy and silent condition [*t*(39) = 0.84, *p* = 0.40; *d* = 0.13]. For both panels, averaged fat scores for each condition are given in Fig 4.

A cross-tabulation of dichotomized per-sample-averaged panel scores (threshold for “deviant”: score ≥ 2) revealed that 32 out of 40 samples (80%) were rated similarly in both conditions by the university panel; when a higher threshold for dichotomization is used (≥ 3, i.e. more severe deviation, perfect agreement between noisy and silent conditions is observed. Also for the abattoir panel the binary fat scores (0, 1) were averaged using thresholds of 0.5 and 0.8, these were then dichotomized again to compare the classification agreement of noisy and silent conditions. The results show that there was a 75% and 90% accordance among the samples from both conditions.

In terms of repeatability of an average panel rating for a given sample no significantly different panel performance was observed for the university panel: ICC3k noise: 0.79 [95% CI: 0.67, 0.87] vs. silent: 0.80 [95% CI: 0.69, 0.88]. Nor was the repeatability impaired for the abattoir panel under noise ICC3k = 0.64 [95% CI: 0.44, 0.78] vs. silent conditions ICC3k = 0.71 [95% CI: 0.56, 0.83]. The latter results, especially with respect to the confidence intervals, have to be viewed with caution because ICC3k is actually defined for quantitative data, not binary.

## Discussion

### 5.1 Effect of non-verbal noise on psychophysical tests

Our results are in partial contrast to Walliczek-Dworschak et al., where nonverbal noise of 70 dB impaired the overall TDI scores of human olfactory functionality whereas no significant impairment of the individual subtests was observed. Walliczek-Dworschak et al. documented a higher impact of nonverbal noise on odor detection thresholds and the identification task compared to the odor discrimination task [12]. Our results confirm the impact on identification task and also a slightly impaired odor detection threshold given the effect size *d*. Seo et al. 2011 found that verbal noise impaired the odor discrimination task more strongly compared to nonverbal noise because the latter interferes more strongly with cognitive processes [11]. Hence we assume that this differential effect accounts for our findings that constant abattoir noise does have little effect on the olfactory performance. Spence et al. suggested as explanation for this phenomenon that humans can selectively give their attention to olfaction, as in his study subjects responded more rapidly when the target was presented in the expected rather than the unexpected modality [23]. Our results demonstrate that trained assessors for a sensory quality control task are indeed able to focus on olfactory stimuli.

According to the linear mixed models, the university panel performed significantly better than the abattoir panel for all psychophysical tests (p < 0.05). This is likely due to the fact that the slaughterhouse panel is not too well accustomed to the olfactory tests which had been commonly applied by the university panel for recruitment and monitoring purposes. Another possible reason is the selection process of the assessors. Presumably, the olfactory performance criteria were stricter for the university panel than for the abattoir panel. Contrary to the researchers’ expectations, abattoir noise did not significantly influence any of the olfactory tests according to the mixed model analysis. Nor was a significant interaction effect observed between noise and accustomation to it.

According to Gudziol and colleagues, the inter-day standard deviation of olfactory thresholds is in the range of 2 to 3 binary dilution steps, just as the slight differences found here are, too, well within that range [24]. For the substances used here, a similar variability of the repeated threshold task was reported for androstenone (*SD* = 2.2) and skatole (*SD* = 2.3) using paper strips [25].

### 5.2 Effect of non-verbal noise on sensory quality control of boar fat samples

To the best of our knowledge no studies evaluating the effect of noise on a complex sensory quality control task have ever been carried out. Furthermore, previous studies used naïve subjects instead of trained assessors as in this study. The effects of auditory cues on chemosensory perception were not consistently taken into considerations in previous studies. In past studies different kinds of noises were used. Seo and colleagues documented a significant influence of acoustic stimuli on the performance of testers carrying out an odor discrimination task [11]. Both verbal noise (comedian talking) and non-verbal noise (din from people at a crowded party) deteriorated the task performance as compared to the silent condition with the effect being stronger for the verbal noise. This implies that there is a difference between constant noises and verbal noises and their effects on human olfactory performance. Further investigation should also consider different levels of sound pressure, since stochastic resonance can lead to increased sensitivity, at least in tactile perception and sense of vision [26].

## Conclusions

This study raises the question as to whether silence, which is often considered mandatory for sensory tests, is truly essential for good sensory practice. Based on pairwise *t*-test analyses, constant technical noise has a small effect on the olfactory performance of trained assessors. Namely, androstenone detection thresholds were slightly impaired for both assessor groups whereas skatole detection thresholds are deemed not to be affected. Odor identification was significantly impaired under noise for the abattoir panel but not for the university panel. However, according to the mixed model analysis abattoir noise did not significantly influence any of the olfactory tests nor was a significant interaction effect observed between noise and accustomation to it. For the more complex quality control task using biological samples with varying amounts of androstenone and skatole, neither noise nor of the interaction effect between noise and accustomation were significant.

The latter results indicate that sensory quality control can be conducted with constant technical noise from an abattoir without diminishing assessors’ performance. Nevertheless, the inconclusive findings from odor detection and odor identification tasks call for further investigation using a larger group of assessors. In addition, the results obtained from this study in which assessors wore headphones still need to be confirmed in a noisy manufacturing environment.

Parts of the results were presented on the poster “Is silence mandatory for sensory evaluations?” on the 11^th^ Pangborn Sensory Science Symposium, 23–27 August 2015, Gothenburg (Sweden).

## Supporting information

S1 Data (XLSX)
