# Correction: Noise and accustomation: A pilot study of trained assessors' olfactory performance

**DOI:** 10.1371/journal.pone.0183875

**Published:** 2017-08-22

**Authors:** Johanna Trautmann Mörlein, Lisa Meier-Dinkel, Jan Gertheiss, Daniel Mörlein

The first author’s name is incorrect. The correct name is: Johanna Trautmann Mörlein. The correct citation is: Trautmann Mörlein J, Meier-Dinkel L, Gertheiss J, Mörlein D (2017) Noise and accustomation: A pilot study of trained assessors’ olfactory performance. PLoS ONE 12(4): e0174697. https://doi.org/10.1371/journal.pone.0174697
